# The beneficial effect of probiotics in the prevention of irinotecan-induced diarrhea in colorectal cancer patients with colostomy: a pooled analysis of two probiotic trials (Probio-SK-003 and Probio-SK-005) led by Slovak Cooperative Oncology Group

**DOI:** 10.3389/fonc.2024.1438657

**Published:** 2024-07-22

**Authors:** Michal Mego, Barbora Kasperova, Jozef Chovanec, Radoslav Danis, Maria Reckova, Branislav Bystricky, Peter Konkolovsky, Silvia Jurisova, Stefan Porsok, Vladimir Vaclav, Maria Wagnerova, Marian Stresko, Bibiana Brezinova, Dagmar Sutekova, Sona Ciernikova, Daniela Svetlovska, Lubos Drgona

**Affiliations:** ^1^ Faculty of Medicine, Comenius University and National Cancer Institute, Bratislava, Slovakia; ^2^ Department of Oncology, St. Jacob Hospital, Bardejov, Slovakia; ^3^ Faculty of Medicine, Comenius University, Bratislava, Slovakia; ^4^ Department of Oncology, Regional Cancer Center, Poprad, Slovakia; ^5^ Department of Oncology, Faculty Hospital, Trencin, Slovakia; ^6^ Department of Oncology, Regional Cancer Center, Komarno, Slovakia; ^7^ Department of Oncology, University Hospital Milosrdni Bratia, Bratislava, Slovakia; ^8^ Department of Oncology, East Slovakia Comprehensive Cancer Center, Kosice, Slovakia; ^9^ Department of Oncology, Faculty Hospital, Trnava, Slovakia; ^10^ Department of Oncology, Trebisov Hospital, Trebisov, Slovakia; ^11^ Department of Oncology, University Hospital Martin, Martin, Slovakia; ^12^ Biomedical Research Center, Cancer Research Institute, Slovak Academy of Sciences, Bratislava, Slovakia

**Keywords:** pooled analysis, irinotecan, diarrhea, probiotics, colorectal cancer, beta-glucuronidase

## Abstract

**Background:**

Probiotics could decrease irinotecan-induced diarrhea due to the reduction of intestinal beta-d-glucuronidase activity. This study included a combined analysis of two clinical trials aimed to determine the effectiveness of the probiotics in the prophylaxis of irinotecan-induced diarrhea in metastatic colorectal cancer (CRC) patients.

**Methods:**

This combined analysis included 46 patients with CRC enrolled in the Probio-SK-003 (NCT01410955) and 233 patients from Probio-SK-005 (NCT02819960) starting a new line of irinotecan-based therapy with identical eligibility criteria. Patients were randomized in a ratio 1:1 to probiotic formulas vs. placebo administered for 12 and 6 weeks, respectively. Due to the different durations of study treatments, only the first 6 weeks of therapy were used for analysis.

**Results:**

In total, 279 patients were randomized, including 142 patients in the placebo and 137 participants in the probiotic arm. Administration of probiotics did not significantly reduce the incidence of grade 3/4 diarrhea compared to placebo (placebo 12.7% vs. probiotics 6.6%, p = 0.11). Neither the overall incidence of diarrhea (placebo 48.6% vs. probiotics 41.6%, p = 0.28) nor the incidence of enterocolitis (placebo 4.2% vs. probiotics 0.7%, p = 0.12) was different in the placebo vs. probiotic arm. However, subgroup analysis revealed that patients with a colostomy who received a placebo had a significantly higher incidence of any diarrhea (placebo 51.2% vs. probiotics 25.7%, p = 0.028) and grade 3/4 diarrhea (placebo 14.6% vs. probiotics 0.0%, p = 0.03) compared to the probiotic arm.

**Conclusions:**

This combined analysis suggests that probiotics could be beneficial in the prevention of irinotecan-induced diarrhea in colorectal cancer patients with colostomy.

## Introduction

Diarrhea represents a common condition in cancer patients undergoing chemotherapy that can severely impact the quality of life and treatment outcomes. Chemotherapy-associated diarrhea is a complex condition requiring a proper understanding of its underlying mechanisms and effective strategies for prevention and management (1).

Diarrhea in cancer patients is caused by various factors, primarily triggered by the aggressive nature of cancer and the side effects of therapeutic interventions such as chemotherapy. The gastrointestinal mucosa, a critical barrier protecting the digestive system, becomes susceptible to damage by treatments that disrupt normal cellular processes. Chemotherapy-induced diarrhea, a common manifestation, is characterized by the toxic effects of anticancer drugs on rapidly dividing cells within the intestinal lining. Additionally, alterations in the gut microbiota, inflammation, and the release of various signaling molecules further contribute to the disruption of physiological bowel functions (1).

The use of probiotics in preventing and managing diarrhea is based on both theoretical considerations and the outcomes of numerous clinical trials (2–6). Lactic acid bacteria play a pivotal role in addressing dysbiosis by competing for substrates with pathogenic bacteria, producing bacteriocins, and enhancing transepithelial resistance (7). Their enzymatic activity influences the activation or deactivation of metabolites responsible for inducing diarrhea (8). Moreover, the production of short-chain fatty acids, essential for the well-being of intestinal mucosal cells, further contributes to the anti-diarrheal effects of probiotics (9, 10).

Irinotecan, a topoisomerase I inhibitor widely used in the treatment of various cancers, including colorectal cancer, has been associated with a higher incidence of diarrhea compared to other chemotherapeutic agents (11). This side effect not only poses discomfort to patients but may also lead to dose reductions or interruptions, compromising the efficacy of the treatment. The incidence of irinotecan-induced diarrhea ranges widely, encompassing 60-90%, with severe diarrhea affecting 20-40% of patients. This gastrointestinal complication assumes critical significance in the landscape of morbidity and mortality associated with irinotecan-based chemotherapy. Identified predisposing factors include age exceeding 65 years, an Eastern Cooperative Oncology Group performance status (ECOG PS) of ≥1, and a history of abdominopelvic radiation (11, 12).

**Figure 1:**
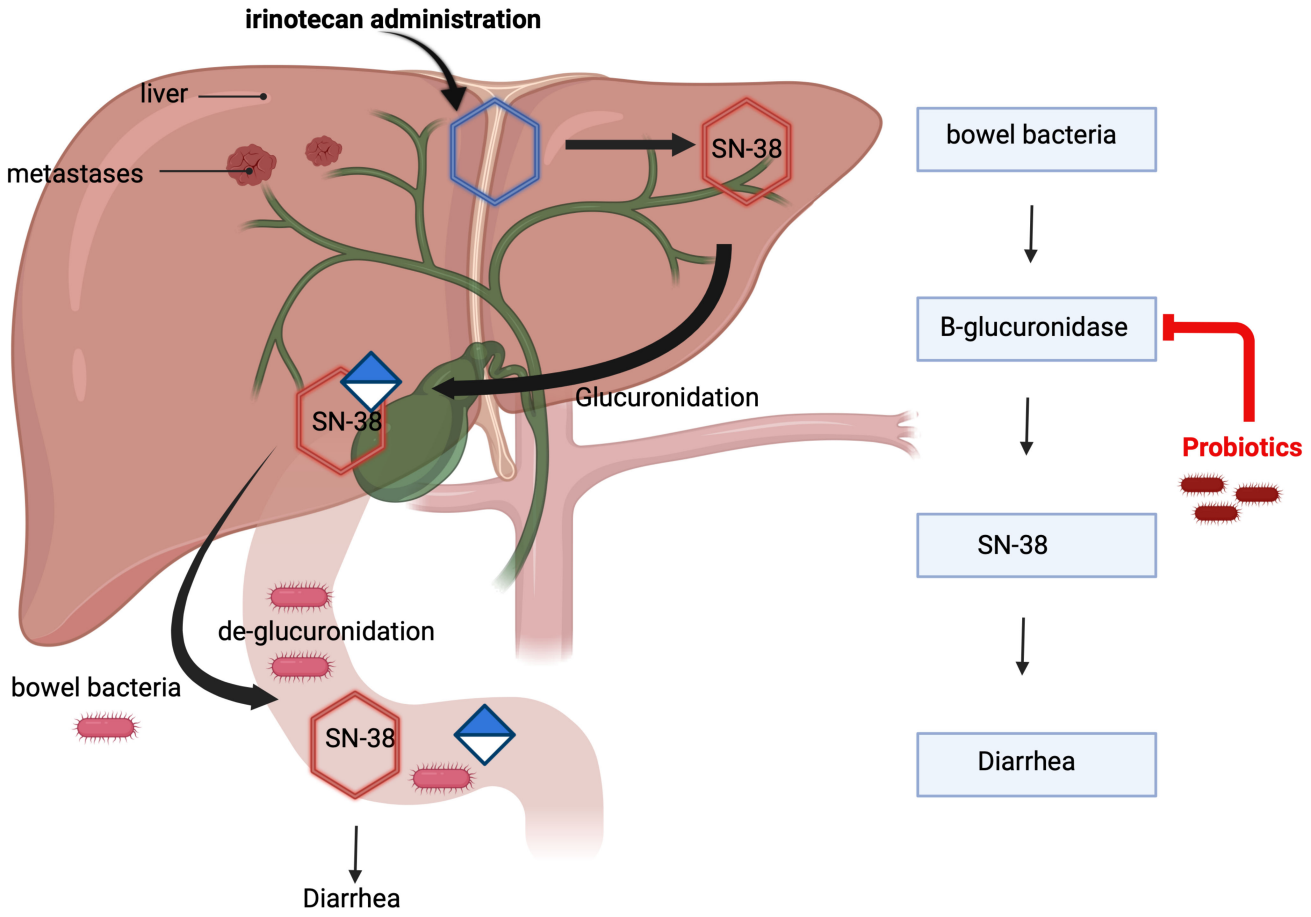
Study hypothesis.

The mechanism of irinotecan-induced diarrhea is mediated by its metabolite SN-38, which is glucuronidated in the liver and subsequently excreted into the intestine. Within the intestinal lumen, bacterial beta-D-glucuronidase deconjugates SN-38, initiating a cascade of events that inflict direct damage to the intestinal mucosa, resulting in malabsorption of water and electrolytes, ultimately culminating in the onset of diarrhea (12). Understanding the intricate mechanisms of irinotecan-induced diarrhea is imperative for devising targeted interventions to enhance the overall management of this chemotherapy-related side effect (13–16). Certain probiotic bacteria have demonstrated the capability to diminish the activity of intestinal beta-D-glucuronidase (14, 15). This suggests a potential avenue for the application of these bacteria in preventing diarrhea in patients undergoing irinotecan-based therapy (**Figure 1**).

Previously, we conducted two clinical trials focused on preventing irinotecan-induced diarrhea in metastatic colorectal cancer patients (17, 18). In the pilot study, which included 46 patients who received the probiotic formula Colon Dophilus™ or placebo, we observed a decreased diarrhea incidence in the probiotic arm with no grade 3/4 diarrhea (17). Based on these results, we performed a phase III trial in the same patient population. In this trial, patients received a combination of *Bifidobacterium* BB-12 and *Lactobacillus rhamnosus* GG, LGG (18). The results of this trial did not confirm the effectivity of probiotics in the prevention of irinotecan-induced diarrhea; however, subgroup analysis suggested their effectivity in patients with colostomy. These trials utilized different probiotic formulas widely available for patients without prescription. The choice of formulas was determined mainly by their availability for investigator-initiated trials from pharmaceutical companies. While Colon Dophilus™ is more complex and contains 10 different probiotic strains, the probiotic formula Probio-Tec^®^ BG-Vcap-6.5 is composed of two strains and has been more widely studied in various clinical scenarios.

The statistical power of subgroup analysis, especially in underrepresented subgroups, is limited in single trials. Taking advantage of identical eligibility criteria and a very similar statistical design of these two clinical trials, we performed pooled analysis aiming to determine the effectiveness of the probiotics in the prophylaxis of irinotecan-induced diarrhea in metastatic colorectal cancer (CRC) patients and identifying specific subgroups that could benefit from preventive administration of probiotics during irinotecan-based chemotherapy. Besides having higher statistical power for the primary endpoint, the dataset of this pooled analysis has increased the number of patients in several specific subgroups compared to individual previous trials, which enables more robust testing, enhances the ability to detect heterogeneity, and improves the generalizability of study results.

## Patients and methods

This combined analysis included two studies; 46 patients with CRC enrolled in the Probio-SK-003 (NCT01410955) between January 2011 and December 2013, starting a new line of irinotecan-based therapy (17) and 233 patients of Probio-SK-005 study (NCT02819960) randomized from March 2016 to May 2022 with identical eligibility criteria as previous trial (18).

### Eligibility criteria

Both trials had the same eligibility criteria (17, 18). Eligible participants were adult patients with histologically proven colorectal cancer starting a new line of chemotherapy based on irinotecan with ECOG PS 0-1 at study entry. Exclusion criteria comprised impossibility to take oral medication, active infection treated by antibiotic therapy, ileostomy or jejunostomy, hypersensitivity to study drug, and any concurrent malignancy other than non-melanoma skin cancer, no other cancer in the past 5 years.

### Trial design

Both trials were multi-centered, double-blinded clinical studies conducted to evaluate the effectiveness of oral probiotic supplements compared to a placebo in preventing severe diarrhea in patients with colorectal cancer who were starting a new round of chemotherapy treatment involving irinotecan. Patients were randomly assigned to receive either the probiotic supplement or the placebo, with an equal number of patients in each group. The randomization process was centralized, where each patient was given a unique identification number and received a corresponding container with the assigned treatment. These containers, indistinguishable from each other, were labeled with sequential numbers assigned randomly to preserve blinding. All researchers, statisticians, and patients remained unaware of which treatment each patient received until the final result analysis.

### Treatment

In Probio-SK-003, the probiotic formula Colon Dophilus™ (produced by Harmoniom International, Inc., Mirabel, Canada) was administered orally at a dose of 3×1cps per day for 12 weeks and each capsule contained 10×10^9^ CFU of bacteria. Whereas, in Probio-SK-005, the probiotic formula Probio-Tec^®^ BG-Vcap-6.5 (produced by Chr. Hansen A/S, Hoersholm, Denmark) containing 2.7x10^9^ CFU was administered orally at a dose of 3x1 cps per day for 6 weeks. No premedication or patient monitoring after probiotic supplementation was required in both trials. The probiotic formula might be taken after meals or snacks to reduce stomach upset. The probiotic formula might be taken after meals or snacks to reduce stomach upset. The capsule should be swallowed whole or opened, and the content mixed with a small amount of food in case of problems with swallowing. Probiotic formula Colon Dophilus™ contained *Bifidobacterium breve HA-129* (25%), *Bifidobacterium bifidum* HA-132 (20%), *Bifidobacterium longum* HA-135 (14.5%), *Lactobacillus rhamnosus* HA-111 (8%), *Lactobacillus acidophilus* HA-122 (8%), *Lactobacillus casei* HA-108 (8%), *Lactobacillus plantarum* HA-119 (8%), *Streptococcus thermophilus* HA-110 (6%), *Lactobacillus brevis* HA-112 (2%), *Bifidobacterium infantis* HA-116 (0.5%). Probio-Tec BG-Vcap-6.5^®^ contained *Bifidobacterium* BB-12 (50%) and *Lactobacillus rhamnosus* GG, LGG (50%).

### Duration of therapy

In Probio-SK-003, the probiotic formula was administered during irinotecan-based chemotherapy for 12 weeks, while in Probio-SK-005, probiotic supplementation lasted for 6 weeks. Due to the different durations of study treatments, only the first 6 weeks of therapy were used for the analysis.

In both trials, patients might also discontinue protocol therapy in the case of intercurrent illness, affecting the patients’ safety in investigator judgment, the ability to deliver treatment or the primary study endpoints, and/or by patient request.

### Concomitant therapy

Patients received full supportive care during the study, including transfusion of blood and blood products, antibiotic treatment, anti-emetics, antidiarrheal agents, analgesics, erythropoietin, or bisphosphonates, when appropriate.

### Treatment evaluation

The clinical assessment encompassed various factors such as demographic information, birthdate, ethnicity, gender, and medical background. This included a detailed account of cancer-specific history, encompassing the date of diagnosis, primary tumor type along with histology findings, past surgical and/or radiological treatments (including dates and specific organ/anatomic regions targeted), current cancer stage, previous systemic therapies, persistent side effects from prior treatments, any history of additional malignancies, and significant medical events within the last six months. The assessment of adverse effects, including diarrhea and enterocolitis, was conducted according to the NCI Common Terminology Criteria for Adverse Events Version 4.1 (CTCAE) (18). Patients maintained diaries to record daily stool frequency and consistency, as well as the use of antidiarrheal medications throughout the study. However, evaluation of patients’ compliance with the prescribed study medications was not performed (17, 18).

### Statistical analysis

Data analysis followed the pre-specified plan for statistical analysis. The patients’ attributes were summarized by presenting the median (range) for continuous variables and frequency (percentage) for categorical variables. The Kolmogorov-Smirnov test was applied to assess the distribution’s normality. If the data followed a normal distribution, sample means were tested using either the Student t-test or analysis of variance (ANOVA), with adjustments like Bonferroni’s or Tamhane’s based on variance homogeneity. For non-normally distributed data, the nonparametric Mann-Whitney U or Kruskal-Wallis H test was utilized. Fisher’s exact test or Chi-square test was employed for categorical data. Event-free survival, specifically concerning diarrhea, was determined utilizing Kaplan-Meier methods, and compared between study arms using the log-rank test. The data were computed from the initiation of probiotic administration (day 1) until the event or the end of the study, at which point the data were censored. All presented p-values are two-sided, with associations considered significant if the p-value was 0.05 or lower. The statistical analyses were conducted using NCSS 2022 statistical software (Hintze J, 2022, Kaysville, UT, USA).

## Results

Patient characteristics and chemotherapy protocols can be found in **Table 1**. There were disparities observed between the groups receiving different treatments. The probiotic arm had a higher proportion of patients with colon cancer compared to rectal cancer, which was in line with previous radiation therapy patterns for rectal cancer. The placebo arm had slightly more patients receiving adjuvant therapy, whereas the probiotic arm had a higher number of patients treated with first-line chemotherapy. Colostomy was slightly more prevalent in the placebo arm. The distribution of irinotecan regimens and other therapies, including 5-FU-based, anti-EGFR, and anti-VEGF therapy, was balanced across both arms.

**Table 1:** Patients’ characteristics.

	Placebo	A	Probiotics	B
	N	%	N	%
**All patients**	142	100.0	137	100.0
Age. median (range)	65 (36-82)		64 (29-82)	
Gender				
male	83	58.5	81	59.1
female	59	41.5	56	40.9
Tumor localization				
colon	86	60.6	96	70.1
rectum	53	37.3	40	29.2
Surgery of primary tumor				
no	28	19.7	36	26.3
yes	112	78.9	101	73.7
Colostomy				
no	101	71.1	102	74.5
yes	41	28.9	35	25.5
Previous radiotherapy to rectum				
yes	34	23.9	22	16.1
no	108	76.1	115	83.9
Previous therapy				
adjuvant chemotherapy	56	39.4	41	29.9
chemotherapy for metastatic disease	79	55.6	70	51.1
5-Fluorouracil- based including capecitabine	75	52.8	63	46.0
anti-VEGF	33	23.2	35	25.5
anti-EGFR	11	7.7	10	7.3
Current therapy				
Line of therapy				
1^st^ line	63	44.4	67	48.9
2^nd^ line	70	49.3	62	45.3
3^rd^ line	9	6.3	6	4.4
4^th^ line	0	0.0	2	1.5
Chemotherapy				
irinotecan weekly	32	22.5	34	24.8
irinotecan every 2 weeks	88	62.0	83	60.6
irinotecan every 3 weeks	22	15.5	20	14.6
5-Fluorouracil	73	51.4	66	48.2
5-Fluorouracil bolus	34	23.9	71	51.8
5-Fluorouracil continues	47	33.1	51	37.2
Capecitabine	48	33.8	41	29.9
5-Fluorouracil- based chemotherapy	122	85.9	112	81.8
anti-EGFR	19	13.4	16	11.7
anti-VEGF	47	33.1	44	32.1

**Figure 2:**
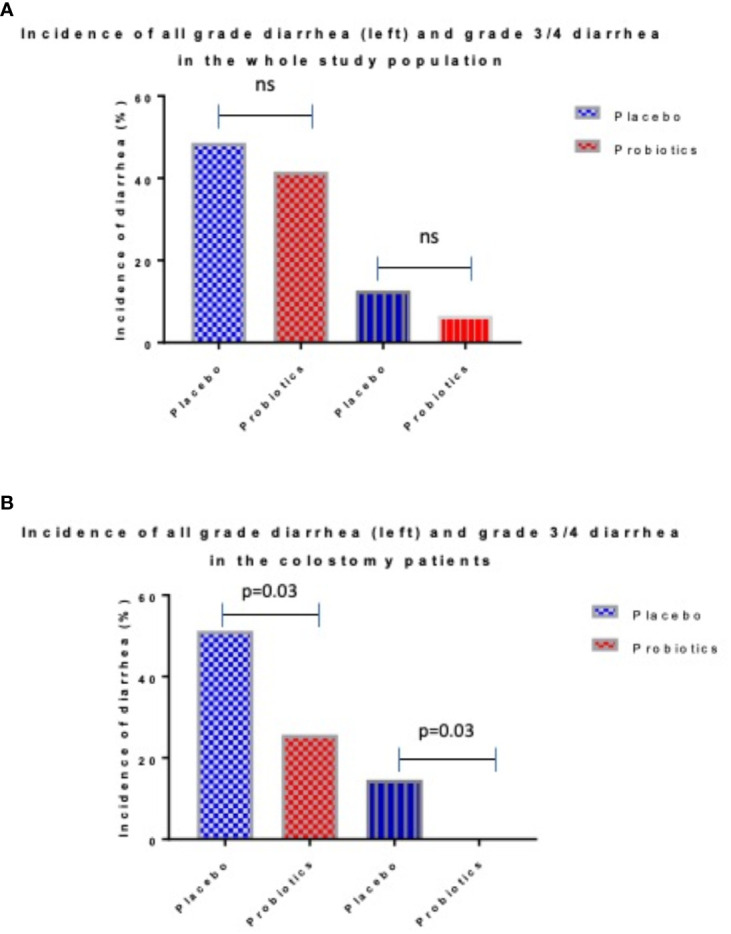
Incidence of diarrhea in whole study populations **(A)** and patients with colostomy **(B)**.

Totally 279 patients were randomized (placebo 142, probiotics 137). Administration of probiotics did not significantly reduce the incidence of grade 3/4 diarrhea compared to placebo (placebo 12.7% vs. probiotics 6.6%, p = 0.11) (**Figure 2A**). Neither the overall incidence of diarrhea (placebo 48.6% vs. probiotics 41.6%, p = 0.28) nor the incidence of enterocolitis (placebo 4.2% vs. probiotics 0.7%, p = 0.12) was different in the placebo vs. probiotic arm (**Table 2**). However, subgroup analysis revealed that patients with a colostomy who received a placebo had a significantly higher incidence of any diarrhea (placebo 51.2% vs. probiotics 25.7%, p = 0.028) and grade 3/4 diarrhea (placebo 14.6% vs. probiotics 0.0%, p = 0.03) compared to the probiotic arm. Moreover, patients with colostomy had no enterocolitis compared to 7.3% of patients in a placebo arm (**Table 3**; **Figure 2B**). Patients in the probiotic arm needed numerically less salvage medication (loperamide) in contrast to the placebo arm (placebo 29.3% vs. probiotics 14.3%, p = 0.17). We did not observe any infection caused by probiotic strains in this study.

**Table 2:** Study results (n=279).

	Placebo	A	Probiotics	B	P-value
Variables	N	%	N	%	
**Diarrhea any grade**	69	48.6	57	41.6	0.28
**Diarrhea grade 3/4**	18	12.7	9	6.6	0.11
Diarrhea (grades)					
0	73	51.4	80	58.4	0.05
1	34	23.9	22	16.1	
2	17	12.0	26	19.0	
3	16	11.3	9	6.6	
4	2	1.4	0	0.0	
**Enterocolitis**	6	4.2	1	0.7	0.12
**Abdominal bloating**	13	9.2	9	6.6	0.5
Patients’ diaries					
mushy stool	112	78.9	112	81.8	0.55
watery stool	75	52.8	76	55.5	0.72
loperamide	32	22.5	31	22.6	1.00
diphenoxylate	34	23.9	24	17.5	0.24
loperamide or diphenoxylate	51	35.9	40	29.2	0.25

**Table 3:** Study results. Colostomy patients only (n=76).

	Placebo	A	Probiotics	B	
Variables	N	%	N	%	P-value
**Diarrhea any grade**	21	51.2	9	25.7	0.03
**Diarrhea grade 3/4**	6	14.6	0	0.0	0.03
Diarrhea (grades)					
0	20	48.8	26	74.3	0.008
1	9	22.0	3	8.6	
2	6	14.6	6	17.1	
3	6	14.6	0	0.0	
4	0	0.0	0	0.0	
**Enterocolitis**	3	7.3	0	0.0	0.24
**Abdominal bloating**	7	17.1	3	8.6	0.33
Patients’ diaries					
mushy stool	33	80.5	28	80.0	1.00
watery stool	26	63.4	19	54.3	0.49
loperamide	12	29.3	5	14.3	0.17
diphenoxylate	10	24.4	7	20.0	0.78
loperamide or diphenoxylate	17	41.5	8	22.9	0.32

## Discussion

In this pooled analysis, the administration of probiotics did not yield statistically significant reductions in grade 3/4 diarrhea, overall diarrhea incidence, or enterocolitis compared to the placebo group. However, a subgroup analysis identified a benefit for patients with colostomy receiving probiotics, showing significantly lower incidences of any diarrhea and grade 3/4 diarrhea compared to the placebo group. Patients with colostomy in the probiotic arm also had no cases of enterocolitis, in contrast to 7.3% in the placebo arm. Additionally, patients in the probiotic arm required numerically less salvage medication (loperamide) than those in the placebo arm. Importantly, no infections were observed related to the probiotic strains used in the study.

Animal models focusing on irinotecan administration have revealed shifts in microbiota composition, marked by increased presence of intestinal *Enterobacteriaceae* spp. and *Clostridium* cluster XL, accompanied by heightened pro-inflammatory cytokines and alterations in mucosa composition leading to reduced adhesion sites (19, 20). These changes contribute to a decrease in symbiotic bacteria and an increase in opportunistic pathogens. While numerous preclinical data suggest the potential benefits of probiotics in mitigating irinotecan-induced gastrointestinal toxicity, clinical evidence remains limited (21–23). A prospective observational trial hints at the ameliorative effects of *Lentilactobacillus kefiri* LKF01 (Fefibios^®^) on severe irinotecan-induced diarrhea in cancer patients (24). Conversely, a phase II/III, randomized, double-blind, placebo-controlled study failed to meet its primary endpoint of reducing grade 3/4 irinotecan-induced diarrhea using a high-concentration multi-strain probiotic supplement (25). This observation aligns with our trials (17, 18). The disparity underscores the complexity of translating preclinical findings into clinical efficacy and emphasizes the need for further investigation into the optimal probiotic strategies for managing irinotecan-induced diarrhea.

Both these trials had the same eligibility criteria, which enabled data pooling. Due to the different durations of study treatment, only the first 6 weeks of therapy were used for this analysis. Differences in outcome in each trial could be related to the different probiotic formulas used as well as different incidences of diarrhea in control arms, which could be related to better management of irinotecan toxicity in the last years. Despite these differences, both trials consistently showed the most pronounced effect of probiotics in the prevention of diarrhea in patients with a colostomy (17, 18). There was no overlap in any probiotic strain used in these clinical trials. However, both formulas contained *Lactobacillus* and *Bifidobacterium*, which are widely utilized in numerous probiotic products, thus increasing the generalizability of study results. Taking into account the results of a similar trial published in the abstract form (25), we suggest that the efficacy of probiotics in reducing irinotecan-induced diarrhea in the unselected patient population is unlikely. These results can’t exclude the potential beneficial effect of gut microbiome modification by other probiotic formulas and/or fecal microbiota transplantation in the study patient population treated with irinotecan-based chemotherapy. Unfortunately, any of the utilized probiotic formulas underwent preclinical testing in animal models of irinotecan-induced diarrhea, which could also affect study results. Future studies assessing any other intervention to modify the gut microbiome composition should incorporate preclinical testing before proceeding to a clinical setting.

In our analysis, the administration of probiotics was associated with a significantly reduced incidence of diarrhea in colostomy patients. We can’t assess if this could be related to a decrease in bowel beta-glucuronidase activity due to probiotics and/or if this is achieved by another mechanism. While the incidence of diarrhea in colostomy patients on the placebo arm and/or grade ¾ diarrhea was not different compared to the whole study population, this was dramatically reduced on the probiotic arm. While shorter bowel length may be a contributing factor, it is also possible that differences in microbiome composition could be influencing this observation. To better understand this phenomenon, future studies should investigate the pre- and post-treatment composition of the gut microbiota, as well as measure beta-glucuronidase activity. Animal models showed that the microbiome composition in colostomy is different compared to normal bowel (26). In a rat model with left colostomy, a significant impact on the growth curve of rats was observed. Analysis of the intestinal microbiota indicated that colostomy primarily influenced the cecal microbiota rather than the colonic microbiota. Notably, there was an increase in the number of enterococci in both the ileum and cecum and elevated levels of cecal lactobacilli, contributing to the promotion of lactic acid bacteria in colostomized rats. Interestingly, there were no substantial differences in the translocation of intestinal bacteria to internal organs (spleen, kidneys, lungs, or liver) among colostomized, laparotomized, and control rats, regardless of their diet. The administration of heat-killed *Lactobacillus acidophilus* strain LB (inactive probiotic bacteria) exhibited a tendency to stimulate bifidobacteria, potentially influenced by culture-medium fermentation substances in the pharmaceutical product. However, this stimulatory effect was abolished by laparotomy and colostomy. Additionally, a trend towards a probiotic-like effect, unaffected by colostomy, was observed, as counts of lactobacilli tended to increase in both the cecum and colon of all animals fed with *Lactobacillus acidophilus* LB (26).

In CRC patients with colostomy, differences in microbial composition were observed as well, showing a reduction in anaerobic bacteria, notably affecting *Alistipes, Akkermansia, Intestinimonas*, and methane-producing archaea. Gene function analysis indicated an underrepresentation of methane and short-chain fatty acid production in patients with a stoma. Moreover, the presence of a stoma correlated with overall decreased taxonomic diversity but increased diversity in the KEGG ((Kyoto Encyclopedia of Genes and Genomes) pathway. Based on the results, patients with a stoma exhibit diminished levels of beneficial microbes for cancer immunotherapy. This study underscores that a stoma can significantly alter both taxonomic and functional profiles in fecal microbiota, emphasizing its potential as a confounding factor in fecal microbiota analyses (27). Accordingly, patients with low vs. high-output ileostomy displayed differences in microbiota composition, particularly in the percentage of Bacteroidota between the high-output and low-output groups (14.8% vs 0.5%; p=0.01) (28). Another study investigated the effects of a probiotic formula (Ecologic^®^825) on the adult human small intestinal ileostoma microbiota. The findings indicated that supplementation with the probiotic formula reduced the growth of pathobionts, such as *Enterococcaceae* and *Enterobacteriaceae*, and decreased ethanol production. These changes were associated with significant alterations in nutrient utilization and resistance to perturbations. The probiotic-mediated alterations, which coincided with an initial increase in lactate production and a decrease in pH, were followed by a sharp increase in the levels of butyrate and propionate (29).

This pooled analysis, beyond several advantages, has some limitations as well. Firstly, clinical trials utilize different probiotic formulas, contributing to the heterogeneity of trials. Moreover, any of the utilized probiotic formulas underwent preclinical testing in animal models of irinotecan-induced diarrhea. Both trials lack compliance measurement as well as assessment of gut colonization by probiotic formula and/or the measurement of stool beta-glucuronidase activity or another potential biomarker of probiotic efficacy. Despite the pooled analysis of the two trials, the statistical power of several subgroups remains low due to the small sample size of the first trial. However, this analysis enables us to confirm the results of probiotic benefit in patients with colostomy as there was only a trend of benefit in the Probio-SK-005 study (18).

## Conclusions

In conclusion, this combined analysis suggests that probiotics could be beneficial in irinotecan-induced diarrhea prevention in colorectal cancer patients with colostomy. We propose that the preservation of healthy microbiota composition could be the simple, effective, and nontoxic approach to reduce gastrointestinal toxicity of irinotecan-based chemotherapy. Future research should prioritize mechanistic studies to investigate the link between stool beta-glucuronidase activity and the risk of irinotecan-induced diarrhea. It is also essential to evaluate various probiotic formulas and fecal microbiome transfer strategies to reduce the incidence of chemotherapy-associated diarrhea. However, one major challenge is that most current approaches have been one-size-fits-all, neglecting the unique composition of an individual’s original microbiome, its colonization resistance, dietary influences, concomitant medications, and host factors that can all impact the microbiome. To address this complexity, it’s crucial to integrate broad translational research into intervention studies, collecting and characterizing biological samples from various time points to understand the intricate interaction between microbiome modification approaches, biomarkers of change, and clinical endpoints. This will help optimize treatment strategies and improve patient outcomes. Until the availability of new pre- and clinical data in this setting, we suggest that the administration of probiotics formulas containing *Lactobacillus* and *Bifidobacterium* strains in colostomy patients treated with irinotecan-based chemotherapy seems prudent. However, there is no evidence to support the role of probiotic administration in unselected populations aimed at reducing irinotecan-induced diarrhea.

## Data availability statement

The raw data supporting the conclusions of this article will be made available by the authors, without undue reservation.

## Ethics statement

The studies involving humans were approved by Ethical Committee of National Cancer Institute, Bratislava, Slovakia. The studies were conducted in accordance with the local legislation and institutional requirements. The participants provided their written informed consent to participate in this study.

## Author contributions

MM: Conceptualization, Data curation, Formal analysis, Funding acquisition, Investigation, Methodology, Project administration, Resources, Supervision, Validation, Visualization, Writing – original draft, Writing – review & editing. BK: Conceptualization, Methodology, Writing – original draft, Writing – review & editing. JC: Investigation, Writing – original draft, Writing – review & editing. RD: Conceptualization, Funding acquisition, Investigation, Methodology, Writing – original draft, Writing – review & editing. MR: Investigation, Supervision, Writing – original draft, Writing – review & editing. BBy: Investigation, Writing – original draft, Writing – review & editing. PK: Investigation, Writing – original draft, Writing – review & editing. SJ: Investigation, Writing – original draft, Writing – review & editing. SP: Investigation, Writing – original draft, Writing – review & editing. VV: Investigation, Writing – original draft, Writing – review & editing. MW: Investigation, Writing – original draft, Writing – review & editing. MS: Investigation, Writing – original draft, Writing – review & editing. BBr: Investigation, Writing – original draft, Writing – review & editing. DSu: Investigation, Writing – original draft, Writing – review & editing. SC: Investigation, Supervision, Validation, Writing – original draft, Writing – review & editing. DSv: Data curation, Investigation, Methodology, Resources, Supervision, Writing – original draft, Writing – review & editing. LD: Conceptualization, Formal analysis, Investigation, Supervision, Validation, Writing – original draft, Writing – review & editing.
